# (3*R*,4*S*,5*S*,8*S*,10*R*,13*R*)-3-Hy­droxy­kaura-9(11),16-dien-18-oic acid

**DOI:** 10.1107/S1600536812002206

**Published:** 2012-01-25

**Authors:** Karren D. Beattie, Mohan M. Bhadbhade, Donald C. Craig, David N. Leach

**Affiliations:** a Centre for Phytochemistry and Pharmacology, Southern Cross University, Lismore Campus, NSW 2480, Australia; b School of Chemistry, University of New South Wales, Sydney, NSW 2052, Australia

## Abstract

The title compound, C_20_H_28_O_3_, was isolated during our investigation into the chemical composition and pharmacological activity of *Centipeda cunninghamii* (DC.) A. Braun & Asch. (Asteraceae). The enanti­opure compound, a diterpene with a carbon skeleton, is composed of three six- and one five-membered rings in chair, twist-boat, half-chair and envelope conformations, respectively. Each mol­ecule makes one intra- and one inter­molecular O—H⋯O hydrogen bond in the crystal lattice, forming hydrogen-bonded chains along [010]. The absolute configuration of the compound was assigned on the basis of optical rotation measurements.

## Related literature

For the characterization of related kaurane diterpenes, see: Reynolds *et al.* (1991 ▶); Piozzi *et al.* (1972 ▶). For literature on the occurrence of the 3*S* isomer of the title compound isolated from *Ichthyothere terminalis* and *Pseudognaphalium cheiranthifolium*, see: Bohlmann *et al.* (1982 ▶); Mendoza & Urzúa (1998 ▶). For the anti­bacterial activity of the 3*S* isomer, see: Mendoza *et al.* (1997 ▶). For phytopharmacological aspects of *Centipeda cunninghamii*, see: Campbell (1973 ▶); Cribb (1988 ▶); D’Amelio & Mirhom, (1998 ▶); Maiden (1975 ▶); Webb (1948 ▶). For optical rotation data of related compounds, see: Bohlmann *et al.* (1982 ▶); Brieskorn & Pöhlmann (1968 ▶); Reynolds *et al.* (1991 ▶).
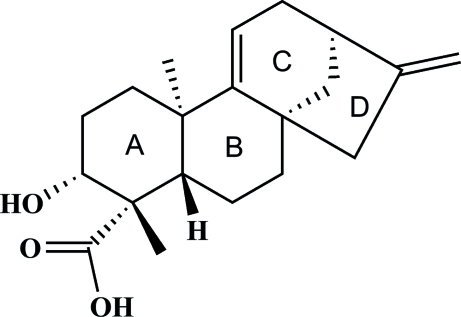


## Experimental

### 

#### Crystal data

C_20_H_28_O_3_
*M* *_r_* = 316.4
Monoclinic, 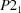

*a* = 8.064 (2) Å
*b* = 10.775 (3) Å
*c* = 10.462 (4) Å
β = 109.70 (2)°
*V* = 855.8 (5) Å^3^
*Z* = 2
Mo *K*α radiation
μ = 0.08 mm^−1^
*T* = 296 K
0.30 × 0.25 × 0.10 mm

#### Data collection

Enraf–Nonius CAD-4 diffractometer
1586 measured reflections
1586 independent reflections
1254 reflections with *I* > 2σ(*I*)
*R*_int_ = 0.000
1 standard reflections every 30 min intensity decay: none

#### Refinement

*R*[*F* ^2^ > 2σ(*F* ^2^)] = 0.044
*wR*(*F* ^2^) = 0.112
*S* = 1.05
1586 reflections
218 parameters
1 restraint
H atoms treated by a mixture of independent and constrained refinement
Δρ_max_ = 0.15 e Å^−3^
Δρ_min_ = −0.17 e Å^−3^

### 

Data collection: *CAD-4 Software* (Enraf–Nonius, 1989 ▶); cell refinement: *CAD-4 Software*; data reduction: *CAD-4 Software*; program(s) used to solve structure: *SHELXS97* (Sheldrick, 2008 ▶); program(s) used to refine structure: *SHELXL97* (Sheldrick, 2008 ▶); molecular graphics: *ORTEP-3 for Windows* (Farrugia, 1997 ▶) and *Mercury* (Macrae *et al.*, 2008 ▶); software used to prepare material for publication: *publCIF* (Westrip, 2010 ▶).

## Supplementary Material

Crystal structure: contains datablock(s) global, I. DOI: 10.1107/S1600536812002206/qk2025sup1.cif

Structure factors: contains datablock(s) I. DOI: 10.1107/S1600536812002206/qk2025Isup2.hkl

Additional supplementary materials:  crystallographic information; 3D view; checkCIF report

## Figures and Tables

**Table 1:** Hydrogen-bond geometry (Å, °)

*D*—H⋯*A*	*D*—H	H⋯*A*	*D*⋯*A*	*D*—H⋯*A*
O3—H1*O*3⋯O1^i^	0.82 (5)	1.83 (5)	2.637 (4)	169 (5)
O1—H1*O*1⋯O2	0.96 (7)	1.94 (6)	2.651 (4)	129 (5)

Symmetry code: (i) 
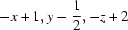
.

**Fig. 1.**
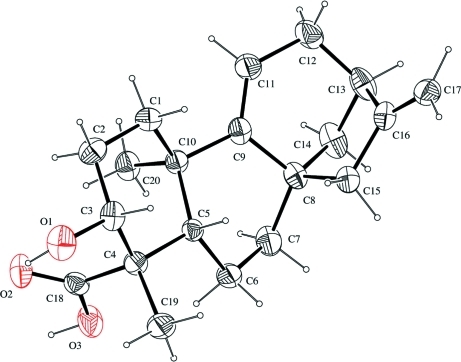
View of (I) with atom numbering scheme. Displacement ellipsoids for non-H atoms are drawn at the 40% probability level.

**Fig. 2.**
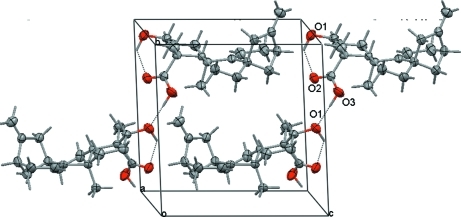
Packing diagram of (I) viewed down the a axis depicting intra and inter molecular O—H···O hydrogen bonding.

## supplementary crystallographic information

### Comment

The title compound C_20_H_28_O_3_ [Fig. 1., systematic name (3*R*,4*S*,5*S*,8*S*,10*R*,13*R*)-3-hydroxykaura-9(11),16-dien-18-oic acid], a diterpene, was isolated during our investigation into the chemical composition and pharmacological activity of active components of *Centipeda cunninghamii*. The herb is native to Australia and New Zealand and has been utilized by the Aboriginals to treat infection (Campbell, 1973; Cribb, 1988; D'Amelio & Mirhom, 1998) and inflammation (Maiden, 1975; Webb, 1948). Compound (I) crystallizes in the monoclinic chiral space group *P*2_1_. The carbon skeleton is composed of three six- and one five-membered rings in chair (ring A), twist-boat (ring B), half-chair (ring C) and envelope conformations (ring D). In the unit cell, two symmetry equivalent molecules make an intramolecular O—H···O bond between the 3-OH (O1) and the carbonyl oxygen (O2) and are linked by intermolecular O—H···O hydrogen bonds involving the carboxylic hydroxyl group (O3, donor) and the 3-OH group (O1, acceptor) into infinite chains extending parallel to the *b* axis (Fig. 2; Table 1).

The majority of the *ent*-kaurane diterpenes characteristically exhibit negative values for optical rotation with the exception of those that feature a double bond between C9 and C11. Piozzi and co-workers (Piozzi *et al.*, 1972) have demonstrated that sequential catalytic hydrogenation of the exocyclic C16—C17 and the endocyclic C9—C11 double bonds in grandiflorenic acid [(4*α*)-kaura-9(11),16-dien-18-oic acid] transforms the optical rotation from +38 to +43° and subsequently to -80° in the saturated product. More recently Reynolds and co-workers (Reynolds *et al.*, 1991) have analysed the solid state and solution characteristics of grandiflorenic acid, and have determined that the introduction of the C9—C11 double bond in grandiflorenic acid has a drastic effect on the molecular conformation and consequently on optical rotation, whereby the *B* ring adopts a boat conformation compared to the regular chair conformation in kaurenoic acid. In the case of compound (I) the observed rotation is [*α*]_D_^22^ + 30.8° (*c* 0.12, MeOH), which is in agreement with values for the methyl ester derivative of the 3*S* isomer, +15° (Bohlmann *et al.*, 1982) and the closely related grandiflorenic acid +32.1° (Brieskorn & Pöhlmann, 1968) and + 46° (Reynolds *et al.*, 1991) and establishes the 3*R*,4*S*,5*S*,8*S*,10*R*,13*R* absolute stereochemistry of compound (I). The 3*S* isomer of compound (I) has been obtained previously from *Ichthyothere terminalis* (Bohlmann *et al.*, 1982) and *Pseudognaphalium cheiranthifolium* (Mendoza & Urzúa, 1998). The antibacterial activity of the 3*S* isomer has also been evaluated and is described in Mendoza *et al.* (1997).

### Experimental

The whole, air-dried flowers of *Centipeda cunninghamii* (100 g) were extracted in 100% ethanol (1 *L*) overnight at ambient temperatures with stirring. The resulting extract was filtered and then partitioned with hexane (3 × 200 ml). The hexane soluble fraction (2.8 g, 2.8% yield *w*/*w*) was evaporated and then subjected to reverse phase preparative HPLC (Gilson preparative HPLC system; Phenomenex Luna C18 column, 5 µm, 50 mm × 21.2 mm; sample loading 50 - 100 mg/injection). The column was eluted using a stepwise gradient of H_2_O/CH_3_CN containing 0.05% CF_3_COOH (3:2 to 1:3 over 16.4 min; 1:3 isocratic for 3.6 min; then 1:3 to 1:9 over 1.0 min; then 1:9 isocratic for 7 minutes) at a flow rate of 15 ml/min. Crystals suitable for X-ray diffraction were obtained by slow evaporation of the H_2_O/CH_3_CN HPLC eluent of (I).

(3*R*,4*S*,5*S*,8*S*,10*R*,13*R*)- 3-Hydroxykaura-9(11),16-dien-18-oic acid (I): Colourless needles (ACN/H_2_O) (26.5 mg, 1.0% yield *w*/*w*, not optimized); [*α*]_D_^22^ + 30.8° (*c* 0.12, MeOH); ^1^H NMR (500 MHz, CD_3_OD): *δ* 5.26 (1*H*, t, *J* = 3.3 Hz, H-11), 4.89 (1*H*, d, *J* = 1.1 Hz, H-17*a*), 4.77 (1*H*, br s, H-17*b*), 3.17 (1*H*, dd, *J* = 4.4, 12.1 Hz, H-3), 2.74 (1*H*, br s, H-13), 2.61 (1*H*, br d, *J* = 14.8 Hz, H-15*a*), 2.44 - 2.40 (1*H*, m, H-12*a*), 2.41 - 2.35 (1*H*, m, H-6a), 2.29 - 2.16 (1*H*, m, H-2a), 2.20 - 2.16 (1*H*, m, H-15*b*), 2.04 (1*H*, m, H-1a), 1.99 - 1.95 (1*H*, m. H-7a), 1.99 - 1.95 (1*H*, m, H-12*b*), 1.93 – 1.85 (1*H*, m, H-6 b), 1.68 - 1.74 (1*H*, m, H-2 b), 1.65 – 1.62 (1*H*, m, H-5), 1.63 - 1.60 (1*H*, m, H-14*a*), 1.51 - 1.47 (1*H*, m, H-7 b), 1.51 - 1.47 (1*H*, m, H-14*b*), 1.38 - 1.32 (1*H*, m, H-1 b), 1.36 (3*H*, s, H-19), 1.10 (3*H*, s, H-20); Lit. (3*S* isomer as the methyl ester derivative, see Bohlmann *et al.*, 1982); ^13^C NMR (126 MHz, CD_3_OD): *δ* 180.1 (C, C-18), 159.6 (C, C-16), 157.3 (C, C-9), 116.3 (CH, C-11), 106.2 (CH_2_, C-17), 79.4 (CH, C-3), 51.5 (CH_2_, C-15), 51.3 (C, C-4), 47.0 (CH, C-5), 46.2 (CH_2_, C-14), 43.6 (C, C-8), 42.8 (CH, C-13), 40.4 (CH_2_, C-1), 39.7 (C, C-10), 39.1 (CH_2_, C-12), 31.0 (CH_2_, C-7), 29.9 (CH_2_, C-2), 24.6 (CH_3_, C-19), 24.4 (CH_3_, C-20), 19.8 (CH_2_, C-6); (+)-LRAPCIMS *m/z* (*rel*. int.): 316 [*M*+H^+^, 0%], 299 (100), 271 (59), 253 (93), 225 (6); (+)-HRAPCIMS *m/z* (*rel*. int): 317.2109 calcd for C_20_H_29_O_3_, 317.2118 (Δ0.0009 a.m.u.).

### Refinement

C-bonded H-atoms were positioned geometrically (C–H = 0.93–0.98 Å) and refined as riding with *U*_iso_(H) = x*U*_eq_(C), where x = 1.5 for methyl and x = 1.2 for the rest. The two O-bonded H-atoms were fully refined. The absolute configuration was assigned by comparison of a similar moiety using NMR spectral data and optical rotation, see section Comment. No Friedel pairs were measured and the refined Flack absolute structure parameter, 1(2), was inconclusive.

### Crystal data

**Table:**

C_20_H_28_O_3_	*F*(000) = 344
*M**_r_* = 316.4	*D*_x_ = 1.228 Mg m^−^^3^
Monoclinic, *P*2_1_	Mo *K*α radiation, λ = 0.71073 Å
Hall symbol: P 2yb	Cell parameters from 11 reflections
*a* = 8.064 (2) Å	θ = 10–11°
*b* = 10.775 (3) Å	µ = 0.08 mm^−^^1^
*c* = 10.462 (4) Å	*T* = 296 K
β = 109.70 (2)°	Block, colourless
*V* = 855.8 (5) Å^3^	0.30 × 0.25 × 0.10 mm
*Z* = 2	

### Data collection

**Table:**

Enraf–Nonius CAD-4 diffractometer	*R*_int_ = 0.0000
Radiation source: fine-focus sealed tube	θ_max_ = 25.0°, θ_min_ = 2.1°
graphite	*h* = −9→9
ω–2θ scans	*k* = 0→12
1586 measured reflections	*l* = 0→12
1586 independent reflections	1 standard reflections every 30 min
1254 reflections with *I* > 2σ(*I*)	intensity decay: none

### Refinement

**Table:**

Refinement on *F*^2^	Secondary atom site location: difference Fourier map
Least-squares matrix: full	Hydrogen site location: inferred from neighbouring sites
*R*[*F*^2^ > 2σ(*F*^2^)] = 0.044	H atoms treated by a mixture of independent and constrained refinement
*wR*(*F*^2^) = 0.112	*w* = 1/[σ^2^(*F*_o_^2^) + (0.0704*P*)^2^] where *P* = (*F*_o_^2^ + 2*F*_c_^2^)/3
*S* = 1.05	(Δ/σ)_max_ = 0.001
1586 reflections	Δρ_max_ = 0.15 e Å^−^^3^
218 parameters	Δρ_min_ = −0.17 e Å^−^^3^
1 restraint	Absolute structure: deduced from optical rotation
Primary atom site location: structure-invariant direct methods	

### Special details

**Table:**

Geometry. All e.s.d.'s (except the e.s.d. in the dihedral angle between two l.s. planes) are estimated using the full covariance matrix. The cell e.s.d.'s are taken into account individually in the estimation of e.s.d.'s in distances, angles and torsion angles; correlations between e.s.d.'s in cell parameters are only used when they are defined by crystal symmetry. An approximate (isotropic) treatment of cell e.s.d.'s is used for estimating e.s.d.'s involving l.s. planes.
Refinement. Refinement of *F*^2^ against ALL reflections. The weighted *R*-factor *wR* and goodness of fit *S* are based on *F*^2^, conventional *R*-factors *R* are based on *F*, with *F* set to zero for negative *F*^2^. The threshold expression of *F*^2^ > σ(*F*^2^) is used only for calculating *R*-factors(gt) *etc*. and is not relevant to the choice of reflections for refinement. *R*-factors based on *F*^2^ are statistically about twice as large as those based on *F*, and *R*- factors based on ALL data will be even larger.

### Fractional atomic coordinates and isotropic or equivalent isotropic displacement parameters (Å^2^)

**Table:**

	*x*	*y*	*z*	*U*_iso_*/*U*_eq_	
O1	0.3554 (4)	0.4597 (3)	0.9863 (3)	0.0658 (8)	
O2	0.4220 (4)	0.2189 (3)	0.9850 (2)	0.0620 (7)	
O3	0.5758 (4)	0.1598 (3)	0.8595 (3)	0.0678 (9)	
C1	0.0447 (4)	0.3543 (4)	0.6459 (4)	0.0521 (10)	
C2	0.1154 (5)	0.3753 (4)	0.7994 (4)	0.0511 (9)	
C3	0.2956 (5)	0.4335 (3)	0.8438 (3)	0.0473 (9)	
C4	0.4293 (4)	0.3558 (3)	0.8032 (3)	0.0395 (8)	
C5	0.3551 (4)	0.3369 (3)	0.6461 (3)	0.0359 (7)	
C6	0.4877 (5)	0.2789 (4)	0.5853 (3)	0.0520 (9)	
C7	0.4047 (5)	0.2222 (4)	0.4458 (3)	0.0572 (10)	
C8	0.2344 (4)	0.2860 (4)	0.3602 (3)	0.0460 (8)	
C9	0.1005 (4)	0.2857 (3)	0.4348 (3)	0.0435 (8)	
C10	0.1677 (4)	0.2779 (3)	0.5907 (3)	0.0399 (8)	
C11	−0.0703 (5)	0.2924 (4)	0.3625 (4)	0.0587 (10)	
C12	−0.1416 (5)	0.3019 (4)	0.2107 (4)	0.0667 (12)	
C13	0.0059 (6)	0.3158 (4)	0.1511 (4)	0.0609 (11)	
C14	0.1503 (6)	0.2238 (4)	0.2209 (4)	0.0645 (11)	
C15	0.2579 (5)	0.4187 (4)	0.3156 (3)	0.0499 (9)	
C16	0.0971 (5)	0.4395 (4)	0.1910 (3)	0.0485 (9)	
C17	0.0429 (5)	0.5449 (4)	0.1297 (4)	0.0567 (10)	
C18	0.4701 (4)	0.2379 (3)	0.8902 (3)	0.0422 (8)	
C19	0.6049 (5)	0.4288 (4)	0.8399 (4)	0.0578 (10)	
C20	0.1658 (5)	0.1410 (4)	0.6329 (4)	0.0518 (9)	
H1A	0.0241	0.4344	0.6009	0.062*	
H1B	−0.0679	0.3121	0.6225	0.062*	
H1O1	0.353 (7)	0.381 (6)	1.029 (6)	0.11 (2)*	
H1O3	0.598 (6)	0.104 (5)	0.916 (5)	0.070 (14)*	
H2A	0.0352	0.4289	0.8251	0.061*	
H2B	0.1212	0.2965	0.8455	0.061*	
H3A	0.2844	0.5133	0.7966	0.057*	
H5	0.3378	0.4215	0.6096	0.043*	
H6A	0.5700	0.3427	0.5799	0.062*	
H6B	0.5545	0.2152	0.6467	0.062*	
H7A	0.3802	0.1353	0.4561	0.069*	
H7B	0.4886	0.2262	0.3977	0.069*	
H11	−0.1504	0.2912	0.4089	0.070*	
H12A	−0.2197	0.3730	0.1847	0.080*	
H12B	−0.2097	0.2281	0.1736	0.080*	
H13	−0.0373	0.3044	0.0524	0.073*	
H14A	0.2340	0.2154	0.1732	0.077*	
H14B	0.1024	0.1429	0.2294	0.077*	
H15A	0.2618	0.4781	0.3863	0.060*	
H15B	0.3652	0.4260	0.2937	0.060*	
H17A	−0.0600	0.5480	0.0549	0.068*	
H17B	0.1071	0.6171	0.1610	0.068*	
H19A	0.6417	0.4530	0.9336	0.087*	
H19B	0.5881	0.5014	0.7839	0.087*	
H19C	0.6936	0.3771	0.8250	0.087*	
H20A	0.0491	0.1080	0.5929	0.078*	
H20B	0.2006	0.1356	0.7300	0.078*	
H20C	0.2464	0.0942	0.6021	0.078*	

### Atomic displacement parameters (Å^2^)

**Table:**

	*U*^11^	*U*^22^	*U*^33^	*U*^12^	*U*^13^	*U*^23^
O1	0.094 (2)	0.0543 (18)	0.0541 (17)	−0.0171 (15)	0.0310 (15)	−0.0191 (14)
O2	0.0852 (18)	0.0558 (16)	0.0539 (14)	0.0077 (16)	0.0353 (14)	0.0151 (14)
O3	0.0799 (19)	0.0664 (19)	0.0635 (19)	0.0311 (16)	0.0324 (16)	0.0300 (16)
C1	0.0394 (19)	0.061 (2)	0.054 (2)	0.0037 (18)	0.0125 (16)	0.0050 (19)
C2	0.053 (2)	0.046 (2)	0.060 (2)	0.0081 (18)	0.0275 (18)	−0.0023 (18)
C3	0.067 (2)	0.0321 (18)	0.047 (2)	−0.0034 (17)	0.0240 (17)	−0.0033 (16)
C4	0.0405 (18)	0.0345 (18)	0.0401 (18)	−0.0065 (15)	0.0094 (14)	−0.0001 (15)
C5	0.0370 (16)	0.0337 (16)	0.0352 (16)	0.0009 (14)	0.0100 (13)	0.0038 (14)
C6	0.0451 (18)	0.066 (2)	0.0468 (19)	0.0127 (19)	0.0179 (16)	0.0072 (19)
C7	0.070 (2)	0.056 (2)	0.052 (2)	0.020 (2)	0.0293 (18)	0.008 (2)
C8	0.055 (2)	0.0439 (19)	0.0368 (17)	0.0057 (18)	0.0120 (15)	0.0002 (16)
C9	0.053 (2)	0.0355 (16)	0.0396 (17)	−0.0086 (17)	0.0127 (15)	−0.0014 (15)
C10	0.0408 (18)	0.0368 (18)	0.0406 (18)	−0.0055 (16)	0.0119 (15)	−0.0012 (15)
C11	0.052 (2)	0.065 (2)	0.051 (2)	−0.020 (2)	0.0080 (18)	0.004 (2)
C12	0.063 (2)	0.070 (3)	0.048 (2)	−0.025 (2)	−0.0063 (19)	0.004 (2)
C13	0.085 (3)	0.054 (3)	0.0337 (18)	0.001 (2)	0.0073 (19)	−0.0051 (16)
C14	0.095 (3)	0.047 (2)	0.047 (2)	0.007 (2)	0.019 (2)	−0.0020 (19)
C15	0.052 (2)	0.055 (2)	0.0442 (19)	−0.0005 (18)	0.0193 (16)	0.0042 (18)
C16	0.059 (2)	0.049 (2)	0.0421 (19)	0.0009 (18)	0.0233 (16)	0.0043 (17)
C17	0.049 (2)	0.058 (2)	0.065 (2)	0.0036 (19)	0.0203 (17)	0.009 (2)
C18	0.0398 (17)	0.0418 (18)	0.0397 (17)	−0.0048 (16)	0.0061 (15)	−0.0008 (16)
C19	0.052 (2)	0.057 (2)	0.058 (2)	−0.017 (2)	0.0090 (18)	0.0025 (19)
C20	0.064 (2)	0.042 (2)	0.046 (2)	−0.0102 (18)	0.0138 (17)	−0.0012 (17)

### Geometric parameters (Å, °)

**Table:**

O1—C3	1.432 (4)	C8—C15	1.536 (5)
O1—H1O1	0.96 (7)	C8—C14	1.538 (5)
O2—C18	1.198 (4)	C9—C11	1.331 (5)
O3—C18	1.312 (4)	C9—C10	1.537 (4)
O3—H1O3	0.82 (5)	C10—C20	1.541 (5)
C1—C2	1.529 (5)	C11—C12	1.499 (5)
C1—C10	1.544 (5)	C11—H11	0.9300
C1—H1A	0.9700	C12—C13	1.525 (6)
C1—H1B	0.9700	C12—H12A	0.9700
C2—C3	1.505 (5)	C12—H12B	0.9700
C2—H2A	0.9700	C13—C16	1.511 (6)
C2—H2B	0.9700	C13—C14	1.516 (6)
C3—C4	1.534 (5)	C13—H13	0.9800
C3—H3A	0.9800	C14—H14A	0.9700
C4—C18	1.532 (5)	C14—H14B	0.9700
C4—C19	1.551 (5)	C15—C16	1.514 (5)
C4—C5	1.562 (4)	C15—H15A	0.9700
C5—C6	1.549 (5)	C15—H15B	0.9700
C5—C10	1.559 (4)	C16—C17	1.305 (6)
C5—H5	0.9800	C17—H17A	0.9300
C6—C7	1.514 (5)	C17—H17B	0.9300
C6—H6A	0.9700	C19—H19A	0.9600
C6—H6B	0.9700	C19—H19B	0.9600
C7—C8	1.527 (5)	C19—H19C	0.9600
C7—H7A	0.9700	C20—H20A	0.9600
C7—H7B	0.9700	C20—H20B	0.9600
C8—C9	1.531 (5)	C20—H20C	0.9600
			
C3—O1—H1O1	105 (4)	C9—C10—C1	109.0 (3)
C18—O3—H1O3	107 (3)	C20—C10—C1	109.5 (3)
C2—C1—C10	114.4 (3)	C9—C10—C5	108.8 (2)
C2—C1—H1A	108.7	C20—C10—C5	112.7 (3)
C10—C1—H1A	108.7	C1—C10—C5	107.9 (3)
C2—C1—H1B	108.7	C9—C11—C12	124.1 (4)
C10—C1—H1B	108.7	C9—C11—H11	118.0
H1A—C1—H1B	107.6	C12—C11—H11	118.0
C3—C2—C1	111.4 (3)	C11—C12—C13	111.5 (3)
C3—C2—H2A	109.3	C11—C12—H12A	109.3
C1—C2—H2A	109.3	C13—C12—H12A	109.3
C3—C2—H2B	109.3	C11—C12—H12B	109.3
C1—C2—H2B	109.3	C13—C12—H12B	109.3
H2A—C2—H2B	108.0	H12A—C12—H12B	108.0
O1—C3—C2	110.8 (3)	C16—C13—C14	102.7 (3)
O1—C3—C4	112.0 (3)	C16—C13—C12	110.4 (3)
C2—C3—C4	112.5 (3)	C14—C13—C12	108.5 (3)
O1—C3—H3A	107.1	C16—C13—H13	111.6
C2—C3—H3A	107.1	C14—C13—H13	111.6
C4—C3—H3A	107.1	C12—C13—H13	111.6
C18—C4—C3	108.6 (3)	C13—C14—C8	101.2 (3)
C18—C4—C19	106.2 (3)	C13—C14—H14A	111.5
C3—C4—C19	108.8 (3)	C8—C14—H14A	111.5
C18—C4—C5	116.5 (3)	C13—C14—H14B	111.5
C3—C4—C5	107.9 (3)	C8—C14—H14B	111.5
C19—C4—C5	108.7 (3)	H14A—C14—H14B	109.4
C6—C5—C10	113.6 (3)	C16—C15—C8	104.0 (3)
C6—C5—C4	114.4 (3)	C16—C15—H15A	111.0
C10—C5—C4	115.1 (2)	C8—C15—H15A	111.0
C6—C5—H5	104.0	C16—C15—H15B	111.0
C10—C5—H5	104.0	C8—C15—H15B	111.0
C4—C5—H5	104.0	H15A—C15—H15B	109.0
C7—C6—C5	114.6 (3)	C17—C16—C13	125.5 (3)
C7—C6—H6A	108.6	C17—C16—C15	126.8 (4)
C5—C6—H6A	108.6	C13—C16—C15	107.7 (3)
C7—C6—H6B	108.6	C16—C17—H17A	120.0
C5—C6—H6B	108.6	C16—C17—H17B	120.0
H6A—C6—H6B	107.6	H17A—C17—H17B	120.0
C6—C7—C8	113.7 (3)	O2—C18—O3	120.7 (3)
C6—C7—H7A	108.8	O2—C18—C4	124.7 (3)
C8—C7—H7A	108.8	O3—C18—C4	114.4 (3)
C6—C7—H7B	108.8	C4—C19—H19A	109.5
C8—C7—H7B	108.8	C4—C19—H19B	109.5
H7A—C7—H7B	107.7	H19A—C19—H19B	109.5
C7—C8—C9	110.5 (3)	C4—C19—H19C	109.5
C7—C8—C15	114.8 (3)	H19A—C19—H19C	109.5
C9—C8—C15	109.7 (3)	H19B—C19—H19C	109.5
C7—C8—C14	112.5 (3)	C10—C20—H20A	109.5
C9—C8—C14	108.7 (3)	C10—C20—H20B	109.5
C15—C8—C14	100.2 (3)	H20A—C20—H20B	109.5
C11—C9—C8	118.8 (3)	C10—C20—H20C	109.5
C11—C9—C10	122.3 (3)	H20A—C20—H20C	109.5
C8—C9—C10	118.9 (3)	H20B—C20—H20C	109.5
C9—C10—C20	108.8 (3)		
			
C10—C1—C2—C3	54.8 (4)	C2—C1—C10—C20	72.3 (4)
C1—C2—C3—O1	176.4 (3)	C2—C1—C10—C5	−50.7 (4)
C1—C2—C3—C4	−57.4 (4)	C6—C5—C10—C9	−55.0 (4)
O1—C3—C4—C18	55.0 (3)	C4—C5—C10—C9	170.5 (3)
C2—C3—C4—C18	−70.5 (3)	C6—C5—C10—C20	65.7 (4)
O1—C3—C4—C19	−60.1 (4)	C4—C5—C10—C20	−68.7 (3)
C2—C3—C4—C19	174.4 (3)	C6—C5—C10—C1	−173.2 (3)
O1—C3—C4—C5	−177.9 (3)	C4—C5—C10—C1	52.3 (4)
C2—C3—C4—C5	56.6 (3)	C8—C9—C11—C12	−0.8 (6)
C18—C4—C5—C6	−67.3 (4)	C10—C9—C11—C12	179.1 (4)
C3—C4—C5—C6	170.3 (3)	C9—C11—C12—C13	−5.6 (6)
C19—C4—C5—C6	52.5 (4)	C11—C12—C13—C16	−67.3 (5)
C18—C4—C5—C10	66.8 (4)	C11—C12—C13—C14	44.5 (5)
C3—C4—C5—C10	−55.6 (3)	C16—C13—C14—C8	42.4 (4)
C19—C4—C5—C10	−173.4 (3)	C12—C13—C14—C8	−74.4 (4)
C10—C5—C6—C7	26.2 (5)	C7—C8—C14—C13	−171.1 (3)
C4—C5—C6—C7	161.1 (3)	C9—C8—C14—C13	66.3 (4)
C5—C6—C7—C8	30.5 (5)	C15—C8—C14—C13	−48.7 (4)
C6—C7—C8—C9	−56.6 (4)	C7—C8—C15—C16	156.9 (3)
C6—C7—C8—C15	68.0 (4)	C9—C8—C15—C16	−78.1 (3)
C6—C7—C8—C14	−178.2 (3)	C14—C8—C15—C16	36.2 (3)
C7—C8—C9—C11	−154.9 (4)	C14—C13—C16—C17	161.6 (4)
C15—C8—C9—C11	77.6 (4)	C12—C13—C16—C17	−82.9 (5)
C14—C8—C9—C11	−31.1 (5)	C14—C13—C16—C15	−19.9 (4)
C7—C8—C9—C10	25.3 (4)	C12—C13—C16—C15	95.6 (4)
C15—C8—C9—C10	−102.2 (3)	C8—C15—C16—C17	167.9 (3)
C14—C8—C9—C10	149.1 (3)	C8—C15—C16—C13	−10.5 (4)
C11—C9—C10—C20	85.3 (5)	C3—C4—C18—O2	−8.6 (4)
C8—C9—C10—C20	−94.8 (4)	C19—C4—C18—O2	108.2 (4)
C11—C9—C10—C1	−34.0 (5)	C5—C4—C18—O2	−130.6 (4)
C8—C9—C10—C1	145.8 (3)	C3—C4—C18—O3	176.3 (3)
C11—C9—C10—C5	−151.5 (4)	C19—C4—C18—O3	−66.9 (4)
C8—C9—C10—C5	28.3 (4)	C5—C4—C18—O3	54.3 (4)
C2—C1—C10—C9	−168.8 (3)		

### Hydrogen-bond geometry (Å, °)

**Table:**

*D*—H···*A*	*D*—H	H···*A*	*D*···*A*	*D*—H···*A*
O3—H1O3···O1^i^	0.82 (5)	1.83 (5)	2.637 (4)	169 (5)
O1—H1O1···O2	0.96 (7)	1.94 (6)	2.651 (4)	129 (5)

Symmetry codes: (i) −*x*+1, *y*−1/2, −*z*+2.
